# Assessment of Mothers' Knowledge and Practices Regarding Oral Rehydration Solution in Sindhuli District, Nepal

**DOI:** 10.7759/cureus.89120

**Published:** 2025-07-31

**Authors:** Rajendra KC, Prabal Tiwari, Sushma Pahadi, Niranjan Chalise, Priyesh KC, Shirish KC

**Affiliations:** 1 Research, Kathmandu University School of Medical Sciences, Dhulikhel, NPL; 2 Medicine, Dhulikhel Hospital, Dhulikhel, NPL; 3 Paediatrics, Kanti Children's Hospital, Kathmandu, NPL; 4 Clinical Research, Doctors on Wheels, Kathmandu, NPL

**Keywords:** diarrhea, knowledge, oral rehydration solution, practice, public health

## Abstract

Background

Oral rehydration solution is a life-saving, cost-effective method in the prevention and management of dehydration in children suffering from diarrhea. Management of dehydration from diarrhea depends on knowledge and practices. Knowledge depends on factors such as educational status and socioeconomic and feeding practices. This study aims to assess the knowledge and practices regarding the use of oral rehydration solutions among mothers in a community within Phikkal Rural Municipality, Sindhuli District, Nepal.

Methods

A community-based cross-sectional study was conducted among 98 mothers using a structured interview schedule containing demographic information, knowledge, and practices. The knowledge section assessed mothers' understanding, and the practice section focused on their actual use. Correct responses in both sections were awarded points. Scores were used to categorize knowledge as adequate or inadequate and practices as good or poor.

Results

The study observed that most mothers had adequate knowledge and good practice. Knowledge level was linked to both ethnicity and educational status. Practice level was significantly associated with educational status. A significant positive correlation was observed between knowledge and practice levels (p=0.006).

Conclusion

Most mothers had good knowledge and practices about oral rehydration solution, though gaps remained in storage and dosing. Community-based education is essential.

## Introduction

Diarrhea is defined as the passage of loose, liquid, or watery stools more than three times a day. The consistency and character of the stool are more important than the number of stools [1]. Globally, diarrheal disease is the second leading cause of death in children under five years of age and is responsible for killing around 525,000 children every year [2]. Oral rehydration therapy is a well-established therapy for the prevention and treatment of dehydration, clinically as effective as intravenous therapy in most cases, and can be carried out at home, thus avoiding hospitalization [3].

Several studies have assessed mothers' knowledge and practices regarding the management of dehydration from diarrhea. Research in Rwanda found that over half of mothers had poor knowledge and practices, with a significant percentage unaware of how to prepare oral rehydration solution (ORS) [4]. A study in Ethiopia showed a mix of good and poor practices [5], while research in India showed varied knowledge levels about ORS preparation, with most mothers having average knowledge [6]. A study conducted in Nepal found that just over half of the mothers had fair knowledge about ORS and fewer could demonstrate good knowledge [7]. However, these studies face limitations like small sample sizes and the challenge of comparing results across different cultural contexts. Studies neglected the factors influencing ORS use, such as healthcare access and community-level education. Studies were unable to include the effectiveness of different educational interventions, the impact of socioeconomic disparities on knowledge and practices, and the barriers to correct ORS preparation and use. Gaps between the knowledge and practices must be filled by awareness and community involvement, which can improve future public health strategies and interventions, leading to better outcomes in child diarrhea management. ORS is widely available in Nepal's rural setting, but there is a gap between knowledge and practices regarding the management of diarrhea. Mothers play a vital role in managing childhood dehydration from diarrhea.

The objective of this study is to explore the association between maternal knowledge and practices regarding ORS in a rural community of Sindhuli District and further examine how these are influenced by sociodemographic factors such as ethnicity and educational status.

## Materials and methods

Study design and location

A cross-sectional research study design was used to study the knowledge and practices regarding ORS among mothers. This research was conducted in Phikkal Rural Municipality, Ward No. 4, of Sindhuli District. Phikkal is a rural municipality located within the Sindhuli District of the Bagmati Province of Nepal. The municipality spans 186.06 square kilometres of area, with a total population of 15,910 according to a 2021 Nepal Census. A non-probability convenience sampling technique was used for this study. Interviews were conducted under conditions of anonymity, using neutral language to promote honest and unbiased responses. Study approval was achieved from the Institutional Review Committee of Kanti Children's Hospital (approval number: 55).

Sample size determination

The total sample size was calculated using Cochran's formula. Based on the prevalence of the study conducted in Nepal, it was 24.3% [7]. The study enrolled mothers of children under the age of five years in Phikkal Rural Municipality, Ward No. 4, Sindhuli District. According to the 2021 Nepal Census, the total population of the municipality was 15,910, with 150 mothers of under-five children. The study included mothers who were willing to participate, i.e., 98, had children under five, and were accessible during the study period. Race and ethnicity were identified through participant self-reports during enrollment interviews. Collecting these data enabled the analysis of demographic patterns in the study population and allowed for the assessment of health equity considerations.

Questionnaire tool and study variables

Practice-related information was collected through a structured questionnaire (see Appendices) using self-reported responses from participants. A structured interview schedule was developed by the researcher herself through an extensive literature search, consulting a research adviser, and subject expertise and colleagues. The instrument was constructed using simple and understandable words in English and Nepali. Questionnaires were developed on the basis of research objectives. 

Scoring criteria for knowledge were "Adequate Knowledge" and "Inadequate Knowledge". For each of the study components, the correct response to the question will be given a score of "1", and the wrong response will be given a score of "0". The maximum score will be 16, and the minimum score will be 0. The scores above 50% will be taken as adequate knowledge, while those below 50% will be taken as inadequate knowledge [1].

Scoring criteria for practice were "Good Practice" and "Poor Practice". For each of the study components, the correct response to the question will be given a score of "1", and the wrong response will be given a score of "0". The maximum score will be 12, and the minimum score will be 0. The scores above 50% will be taken as good practice, while those below 50% will be taken as poor practice [1].

A structured interview schedule was used for data collection. The interview was taken individually. Data collection was conducted during the daytime (10:00 a.m. to 4:00 p.m.). It took about 20-25 minutes to collect data from each respondent. The researcher collected the data of 7-8 respondents per day. At the end of data collection, researchers thanked the respondents for their cooperation and provided informal health education on ORS. The data were collected by the researcher herself over a period of two weeks. All the obtained information was stored in a secure, locked cabinet. Then, researchers entered data into a secure database for analysis. Data entries were verified for accuracy through double-checking. The data was stored on the researcher's personal laptop, secured with a strong password, and regular backups were ensured to an encrypted external drive.

Data analysis

Data analysis was conducted using IBM SPSS Statistics for Windows, Version 20.0 (IBM Corp., Armonk, New York, United States), following data collection. The data was analyzed using descriptive statistics, such as frequency, percentage, mean, and standard deviation. Inferential statistics, i.e., chi-squared tests, were applied to explore the associations between independent and dependent variables.

## Results

A total of 98 (100%) participants from Phikkal Rural Municipality, Ward No. 4, Sindhuli, participated in the study. The mean age of participants was 29.28 years (range: 21-38 years). More than half, 50 (51%), were Brahmin/Chhetri, 37 (37.8%) were Janjati, and 11 (11.2%) were Dalit. Forty-five (45.9%) participants had secondary education, 30 (30.6%) had basic education, and 23 (23.5%) had education above the secondary level. The majority occupation includes agriculture (44, 44.9%), followed by homemaking (32, 32.7%) (Table 1).

**Table 1:** Sociodemographic characteristics of the respondents

Category	Frequency n (%)
Age (years)	
≤30	55 (56.1%)
>30	43 (43.9%)
Ethnicity	
Brahmin/Chhetri	50 (51%)
Janjati	37 (37.8%)
Dalit	11 (11.2%)
Educational status	
Basic education (1-8)	30 (30.6%)
Secondary education (9-12)	45 (45.9%)
More than secondary (13 and above)	23 (23.5%)
Occupation	
Homemaker	32 (32.7%)
Agriculture	44 (44.9%)
Service holder	9 (9.2%)
Business	5 (5.1%)
Teacher	8 (8.2%)

Knowledge regarding ORS

Most participants, 97 (99%), acknowledged that ORS should be given for diarrhea. Around two-thirds, 65 (66.3%), were aware that ORS should be given after three or more loose stools. Ninety-two (93.9%) participants were able to recognize that the use of ORS can manage dehydration from diarrhea. Most, 96 (98%), said that ORS should be given with extra fluids. Only 65 (66.3%) correctly recognized sugar and salt as ORS components. Nearly all 97 (99%) knew that ORS is taken orally and prepared by mixing with clean water. Knowledge of signs indicating when to give ORS and its availability at health posts was also high. However, only 47 (48%) correctly said the role of ORS in replacing lost fluids and electrolytes, and 18 (18.4%) knew that it should be administered in small sips. Knowledge regarding storage duration (24 hours) and appropriate volume for different age groups varied (Table 2).

**Table 2:** Knowledge of participants regarding ORS ORS: oral rehydration solution

Variable	Frequency n (%)
Condition to give ORS: Diarrhea	97 (99%)
ORS should be given to children after experiencing loose stool: Three times or more	65 (66.3%)
Condition prevented by ORS: Dehydration	92 (93.9%)
If a child suffers from diarrhea: ORS and extra fluids should be given	96 (98%)
Major constituents of ORS: Sugar and salt	65 (66.3%)
Means of ORS administration: By drinking	97 (99%)
Method of ORS preparation: ORS is prepared by mixing with water	97 (99%)
Indication: ORS should be given to a child when the child has ​​​​​diarrhea and shows signs of dehydration	79 (80.6%)
Health institute that provides ORS free of cost: Health Post	97 (99%)
Role of ORS: ORS helps a child by providing the body with essential fluids and salts lost during diarrhea	47 (48%)
Children should drink ORS by: Small sips throughout the day	18 (18.4%)
Time to give ORS after diarrhea: ORS should be given to a child with diarrhea within a day of diarrhea	81 (82.7%)
Time duration for storing prepared ORS: 24 hours	43 (43.9%)
Time to administer ORS to a dehydrated child: After every loose stool	55 (56.1%)
Amount of ORS that should be fed to an under-two-year-old child after every loose stool: 50-100 ml	60 (61.2%)
Amount of ORS that should be fed to a child above two years old after every loose stool: 100-200 ml	43 (43.9%)

Practice regarding ORS

Correct practice about the use of cooled boiled water for ORS preparation was reported by 46 (46.9%) participants. A majority, 90 (91.8%), used one whole packet of ORS per liter of water. Proper handwashing before food preparation was practiced by 96 (98%) participants, and continued breastfeeding during diarrhea was reported by 90 (91.8%) of them. About 73 (74.5%) covered the container used for ORS storage. Only 44 (44.9%) reported continuing ORS until the child was fully hydrated (Table 3).

**Table 3:** Practice of participants on ORS preparation ORS: oral rehydration solution

Variables	Frequency n (%)
Type of water used for the preparation of ORS: Cooled boiled water	46 (46.9%)
Amount of water for the preparation of 1 packet ORS: 1 liter	90 (91.8%)
Correct method of mixing ORS sachet in water: By moving a spoon until fully dissolved	54 (55.1%)
Amount of ORS to be mix in water at a time: 1 full packet of ORS	73 (74.5%)
Whether you cover the utensils containing solution or not: Yes	94 (95.9%)
Whether you wash hands with soap and water before preparing ORS or not: Yes	96 (98%)
Whether you continue breastfeeding to child while giving ORS for diarrhea or not: Yes	90 (91.8%)
Action to be carried out if child vomits after drinking ORS: Few minutes should be waited and small sips should be offered	60 (61.2%)
Place to store ORS at home: Cool and dry place	59 (60.2%)
Amount of ORS to be feed to child with diarrhea after passing each loose stool: About equal amount of stool	51 (52%)
Time period to continue ORS after child's diarrhea has stopped: Until the child is fully hydrated	44 (44.9%)
Drinks that should be avoided while treating diarrhea with ORS: Soft drinks and energy drinks	85 (86.7%)

Knowledge and practice scores

Table 4 shows the practice level regarding ORS. The total practice level score was 12. Based on the total score, it was categorized into two levels. Those with scores above 50% were categorized as having a good level of practice, while those with scores less than 50% were categorized as having a poor level of practice. So, in this study, 75 (76.5%) showed a good level of practice, and 23 (23.5%) had a poor level of practice. The mean practice score was 8.59, and the standard deviation was 2.38.

**Table 4:** Participants' practice scores regarding ORS Mean±SD=8.59±2.38 ORS: oral rehydration solution

Level of practice	Frequency n (%)
Good practice (>50%)	75 (76.5%)
Poor practice (<50%)	23 (23.5%)

Table 5 shows the knowledge level regarding ORS. The total knowledge level score was 16. Based on the total score, it was categorized into two levels. Those with scores above 50% were categorized as having an adequate level of knowledge, while those with scores less than 50% were categorized as having an inadequate level of knowledge. So, in this study, 85 (86.7%) showed an adequate level of knowledge, and 13 (13.3%) had an inadequate level of knowledge. The mean knowledge score was 11.55, and the standard deviation was 2.37.

**Table 5:** Participants' knowledge score regarding ORS Mean±SD=11.55±2.37 ORS: oral rehydration solution

Level of knowledge	Frequency n (%)
Good knowledge (>50%)	85 (86.7%)
Poor knowledge (<50%)	13 (13.3%)

Correlation between knowledge, practice, ethnicity, and education

Table 6 shows that the level of knowledge score is statistically associated with ethnicity (p=0.03).

**Table 6:** Correlation between level of knowledge and ethnicity (n=98) f: frequency; χ2: chi square * indicates level of significance <0.05.

Ethnicity	Level of practice		χ2	P-value
	Good f (%)	Poor f (%)	4.683	0.03*
Brahmin/Chhetri	47 (94%)	3 (6%)		
Others (Janjati, Dalit)	38 (79%)	10 (20.8%)		

Table 7 shows that the level of knowledge score is statistically associated with educational status (p=0.01).

**Table 7:** Correlation between level of knowledge and educational status (n=98) f: frequency; χ2: chi square * indicates level of significance <0.05.

Educational status	Level of practice		χ2	P-value
	Good f (%)	Poor f (%)	15.721	0.001*
Basic education	20 (66.7%)	10 (33.3%)		
Secondary education and more than secondary	65 (95.6%)	3 (4.4%)		

There was a statistically significant correlation between knowledge and practice levels (p=0.006), indicating that participants with adequate knowledge were more likely to demonstrate good practice (Table 8).

**Table 8:** Correlation between level of knowledge and practice regarding ORS f: frequency; χ2: chi square; ORS: oral rehydration solution * indicated level of significance <0.05 (n=98).

Level of knowledge	Level of practice		χ2	P-value
	Good f (%)	Poor f (%)	7.700	0.006*
Adequate	6 (6.12%)	7 (7.14%)		
Inadequate	69 (72.6%)	16 (16.32%)		

## Discussion

This study assesses the knowledge and practices regarding ORS among mothers in a community. The results revealed a knowledge-practice gap among mothers concerning the use of ORS; although some understood its role in managing dehydration, many lacked practical knowledge regarding the correct storage duration of ORS and the appropriate frequency or amount of administration during diarrheal episodes.

In this study, nearly half of the mothers were aged between 31 and 35 years, with a mean age of around 29 years. Most participants belonged to the Brahmin and Chhetri ethnicities, and most had secondary-level education. It is well-known that sociodemographic factors, such as education and ethnicity, can influence health practices, which was also similar in our study.

Almost all mothers, 97 (99%), knew when ORS should be given, which was similar to a study from Nigeria where 376 (93.4%) recognized the importance of ORS for managing diarrhea [8]. A significant proportion, 65 (66.3%), also knew that ORS should be administered after three or more episodes of loose stools, comparable to the 96 (60%) found in Rwanda [4]. However, this still leaves a noticeable knowledge gap in about a third of the participants.

Interestingly, while 92 (93.9%) were aware that ORS helps prevent dehydration, this figure was much higher than the 208 (56.3%) reported in another Nigerian study [9]. Differences in health education and literacy levels between study populations might explain this variation.

Over half, 55 (56.1%), knew the correct practice about ORS after each loose stool, which is better than the 100 (34.4%) reported in Ethiopia [5], but still indicates room for improvement. On the other hand, almost all mothers knew that ORS is provided free at health posts, a finding consistent with the study in Kapilvastu, Nepal [7].

Participants were unaware of the use of once-prepared ORS within 24 hours. Only 42 (43.9%) participants answered correctly, which is similar to the findings in the Tamil Nadu study, where only 189 (42%) knew about the proper use [1]. Interestingly, Ethiopian mothers demonstrated higher knowledge, 349 (82.6%), in this regard [10], suggesting differences in health education programs.

Regarding practice, only about half of the mothers, 46 (46.9%), reported using cooled boiled water for ORS preparation. This closely matches a previous Ethiopian study [10]. It was interesting to see that almost all participants, 90 (91.8%), knew how to use one liter of water to prepare one ORS packet, again reflecting findings from Ethiopia [10].

Breastfeeding during diarrhea was continued by 90 (91.8%) mothers in this study, a figure quite similar to a study done in Nagaland [11]. Good hygiene was also noted, with nearly all mothers washing hands with soap and water before ORS preparation, in line with findings from Sunsari, Nepal [12].

Moreover, most mothers, 60 (61.2%), waited and offered small sips if vomiting occurred after ORS intake, similar to observations in Kano state [13].

Overall, 85 (86.7%) mothers were found to have adequate knowledge of ORS. This was higher than the 72% (324) adequate knowledge reported in Tamil Nadu [1] and substantially better compared to the Kapilvastu study, where only 24.3% (53) of mothers had good knowledge [7]. 

Notably, a significant association was found between knowledge and ethnicity (p=0.041), although limited literature was available to support this observation. In addition, there was a strong association between mothers' education level and their knowledge (p=0.001). Educational status was also significantly associated with practice (p=0.001), matching results from Ethiopia [10].

Finally, the study revealed a significant correlation between knowledge and practice (p=0.05), consistent with similar findings in a Nigerian study [14]. This suggests that enhancing mothers' knowledge through targeted health education programs could directly improve their ORS-related practices.

The results, however, showed a gap, particularly regarding ORS storage and administration, which need to be addressed through community health awareness programs.

Limitations

The study was conducted in a single setting with a limited sample size, so the findings cannot be generalized to other settings. Since the researcher introduced herself as a healthcare professional, mothers might have provided information that was desirable for the researcher to hear. Additionally, recall bias was possible since the participants were asked to recall information on how they had prepared ORS in the past. One other limitation of this study is the reliance on self-reported data to assess practice levels.

## Conclusions

Based on the findings of the study, most of the participants have adequate level of knowledge and good practice regarding ORS as majority of the participants knew that ORS should be given to the child after experiencing loose stool for three times or more, almost all participants knew that dehydration can be prevented by ORS, most of them used one liter of water for the preparation of one packet of ORS, and most of them continued breastfeeding during diarrhea and washed hands with soap and water before preparing ORS. Knowledge about the role of ORS, the amount of ORS to feed, and the correct way of giving ORS to children is insufficient and needs to be addressed.

Practice regarding the type of water used for the preparation of ORS and the time period to continue ORS after a child's diarrhea has stopped is poor and needs to be addressed. This study shows a statistically significant association between the level of knowledge regarding ORS with ethnicity and the educational status of mothers and a statistical association between the level of practice regarding ORS and the educational status of mothers. This study also shows the statistically significant association between the level of knowledge regarding ORS and the level of practice.

## Figures and Tables

**Figure 1:**
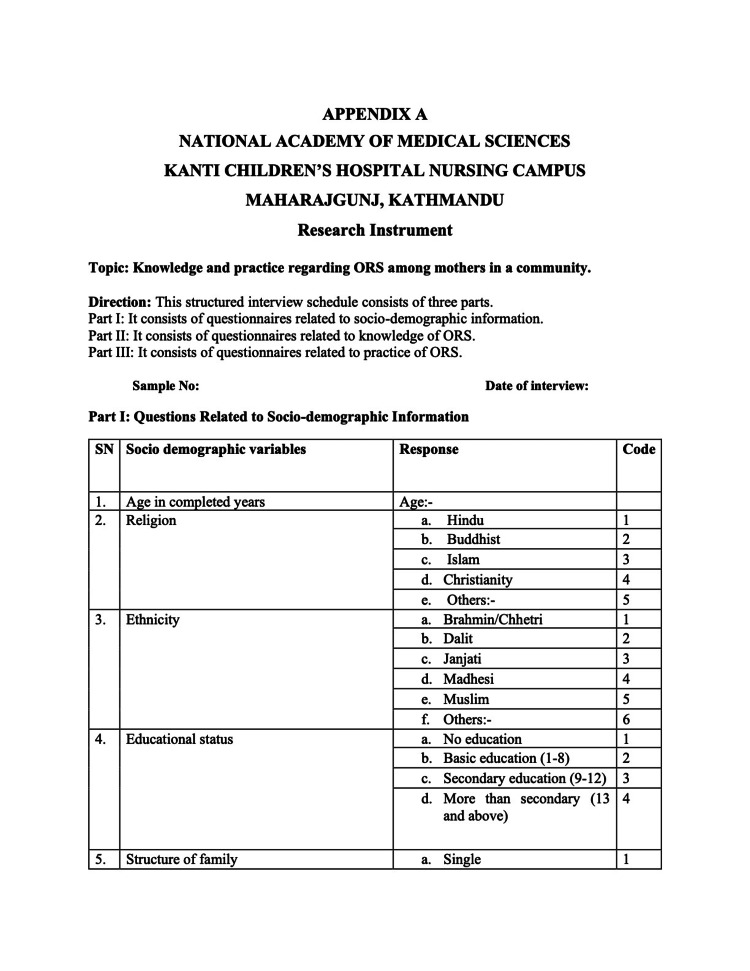
English version of the questionnaire

**Figure 2:**
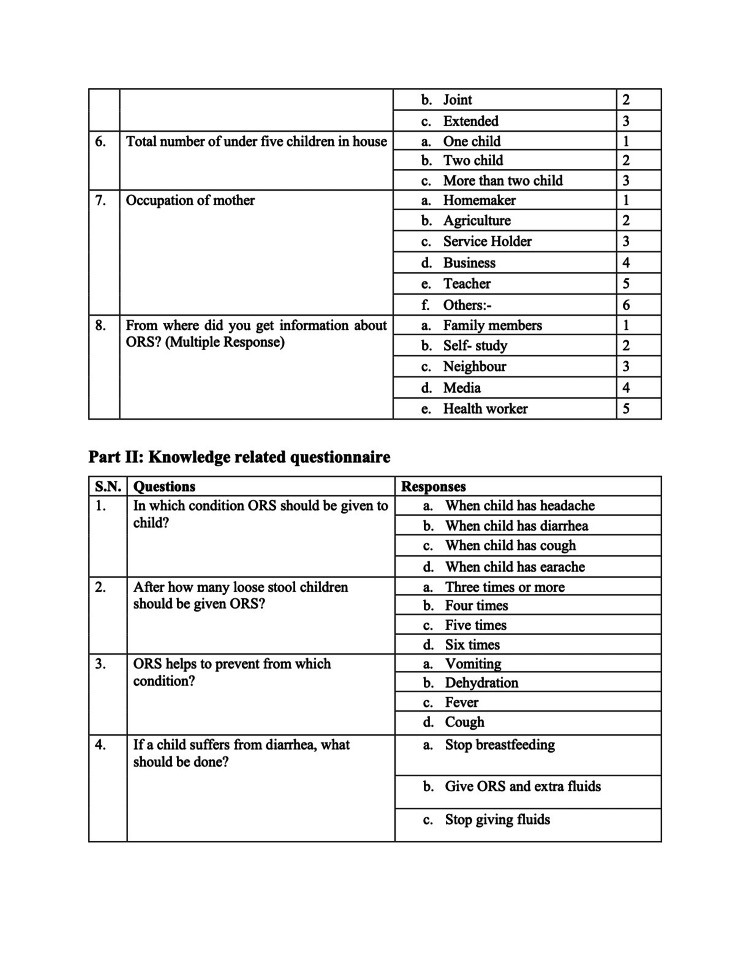
English version of the questionnaire (continuation)

**Figure 3:**
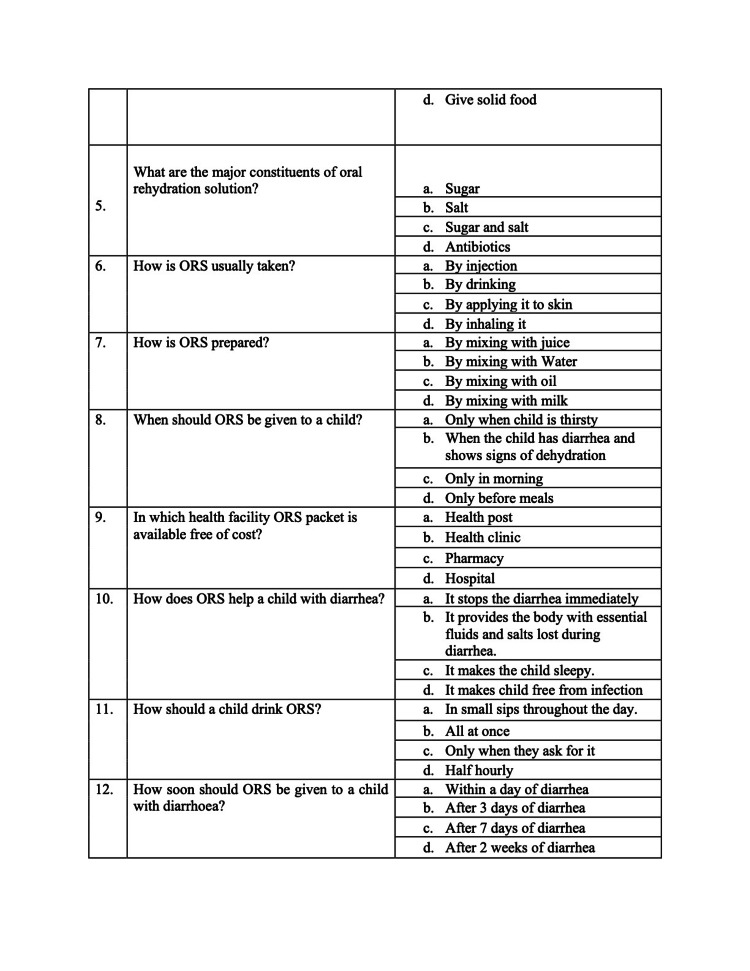
English version of the questionnaire (continuation)

**Figure 4:**
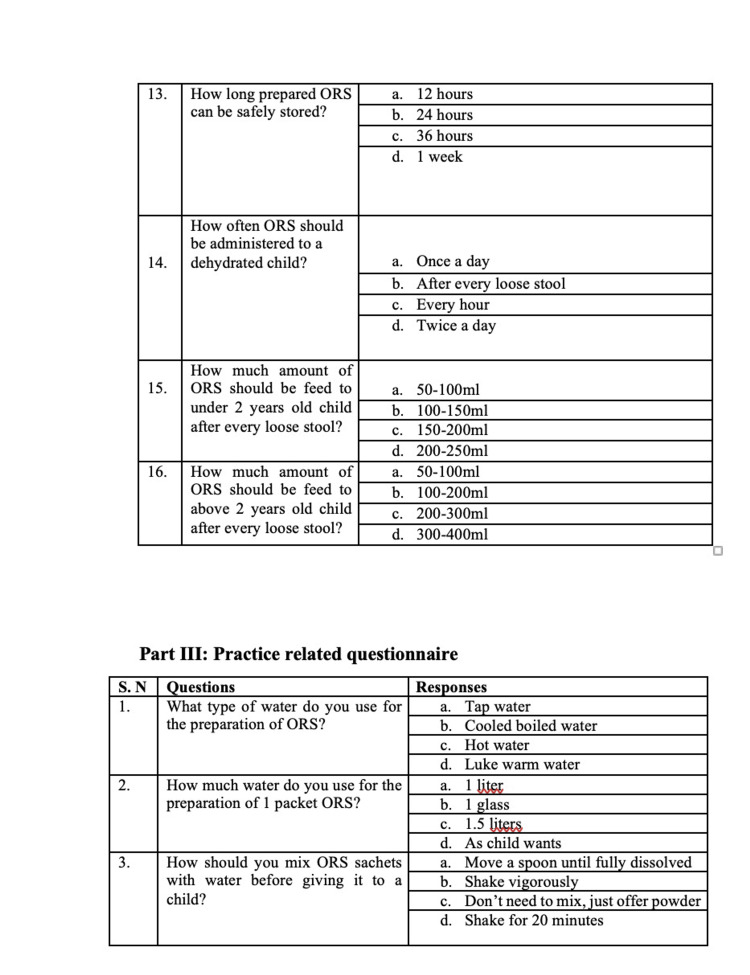
English version of the questionnaire (continuation)

**Figure 5:**
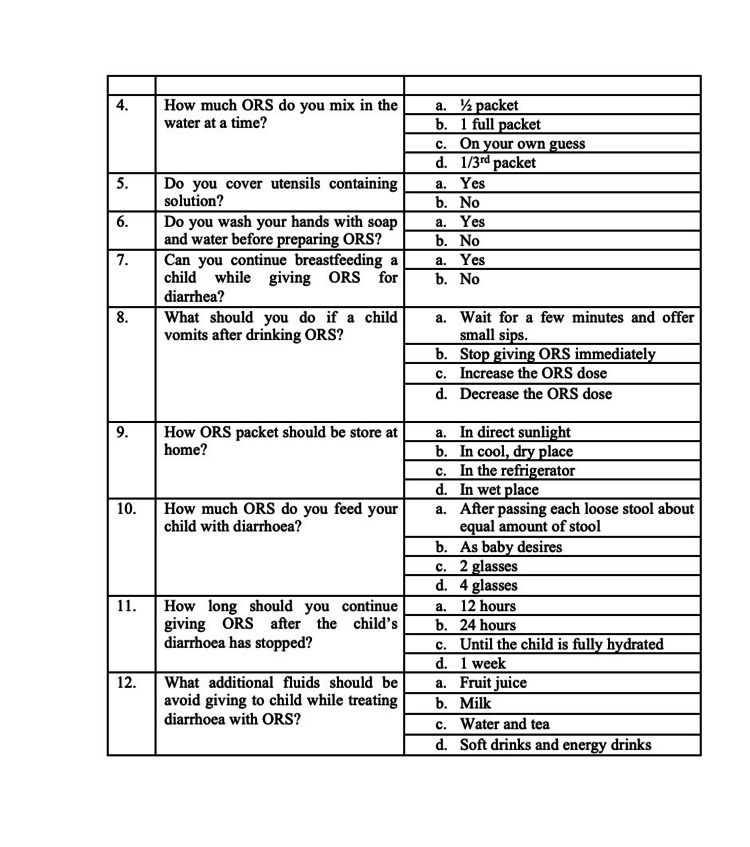
English version of the questionnaire (continuation)

**Figure 6:**
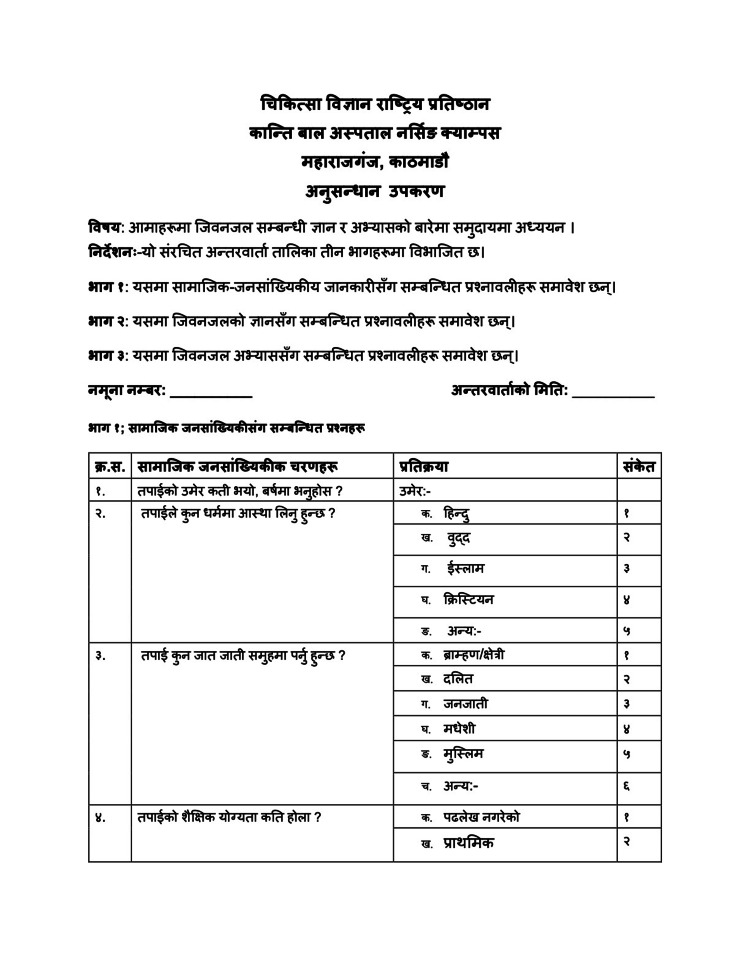
Nepali version of the questionnaire

**Figure 7:**
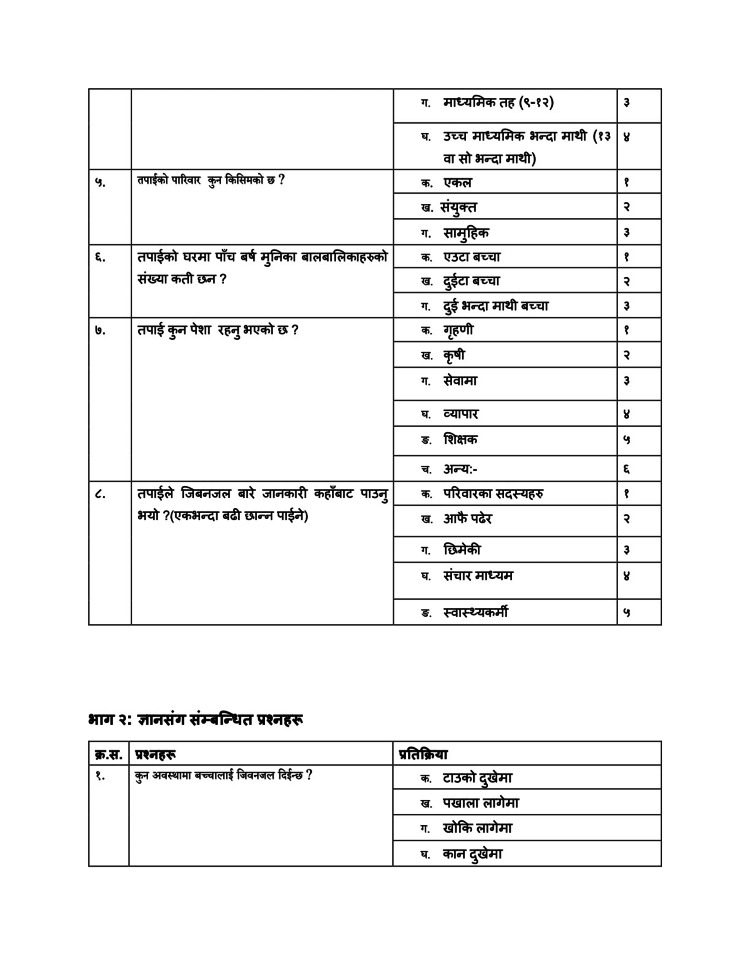
Nepali version of the questionnaire (continuation)

**Figure 8:**
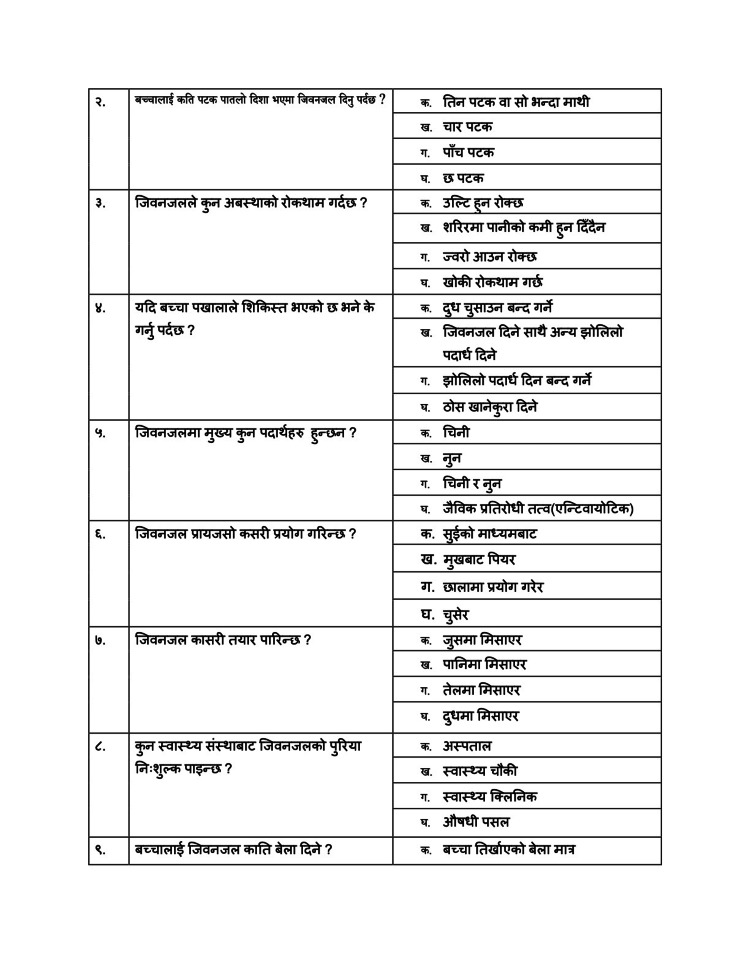
Nepali version of the questionnaire (continuation)

**Figure 9:**
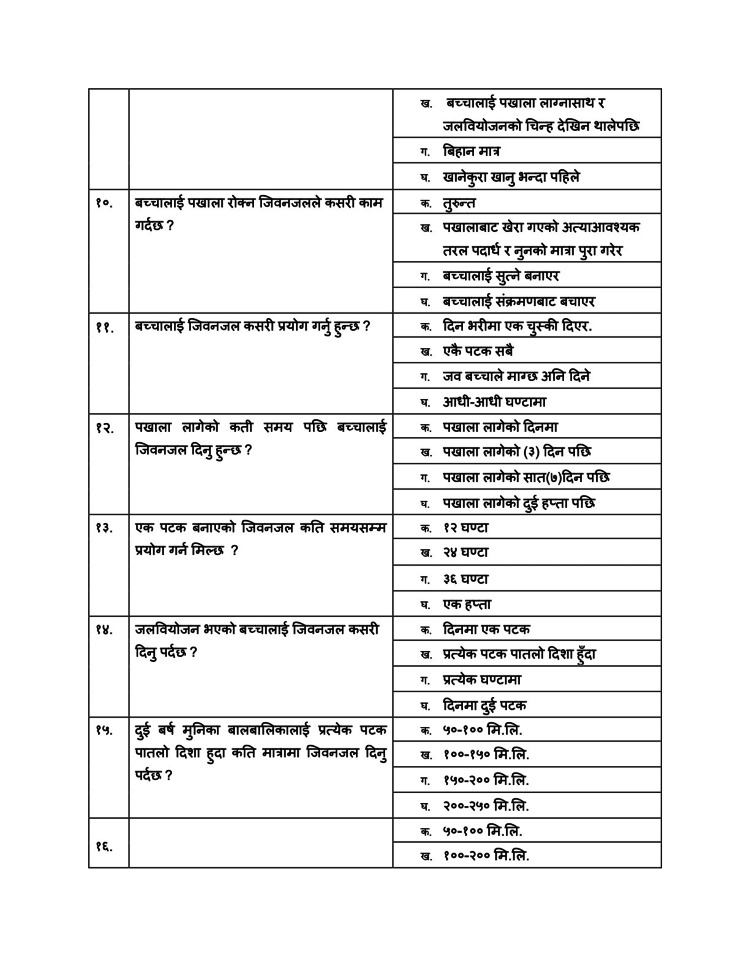
Nepali version of the questionnaire (continuation)

**Figure 10:**
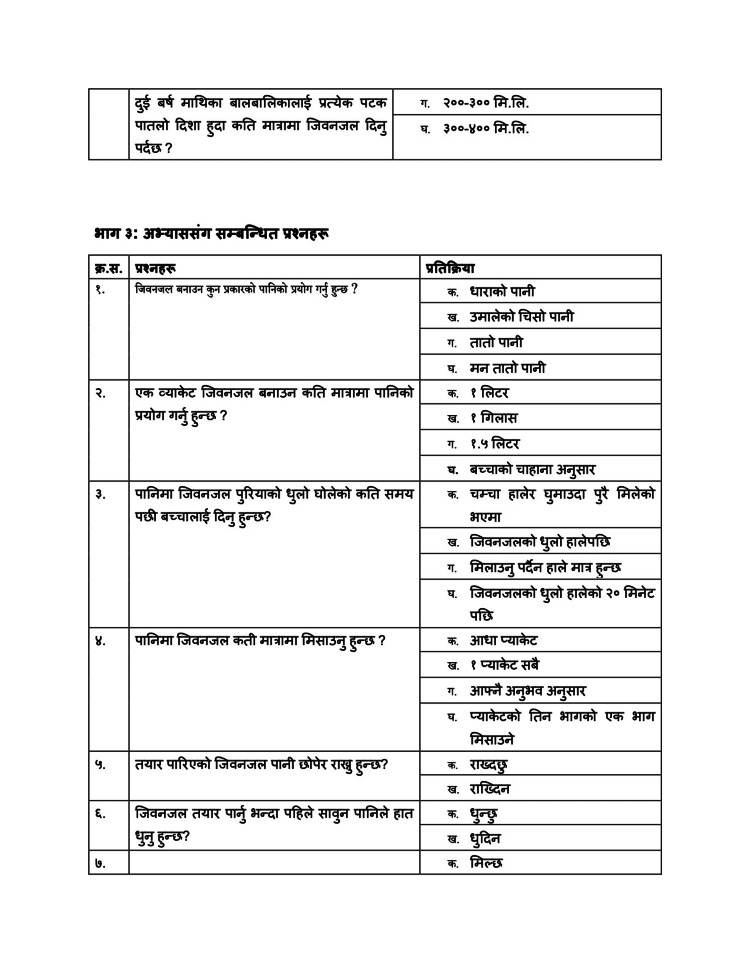
Nepali version of the questionnaire (continuation)

**Figure 11:**
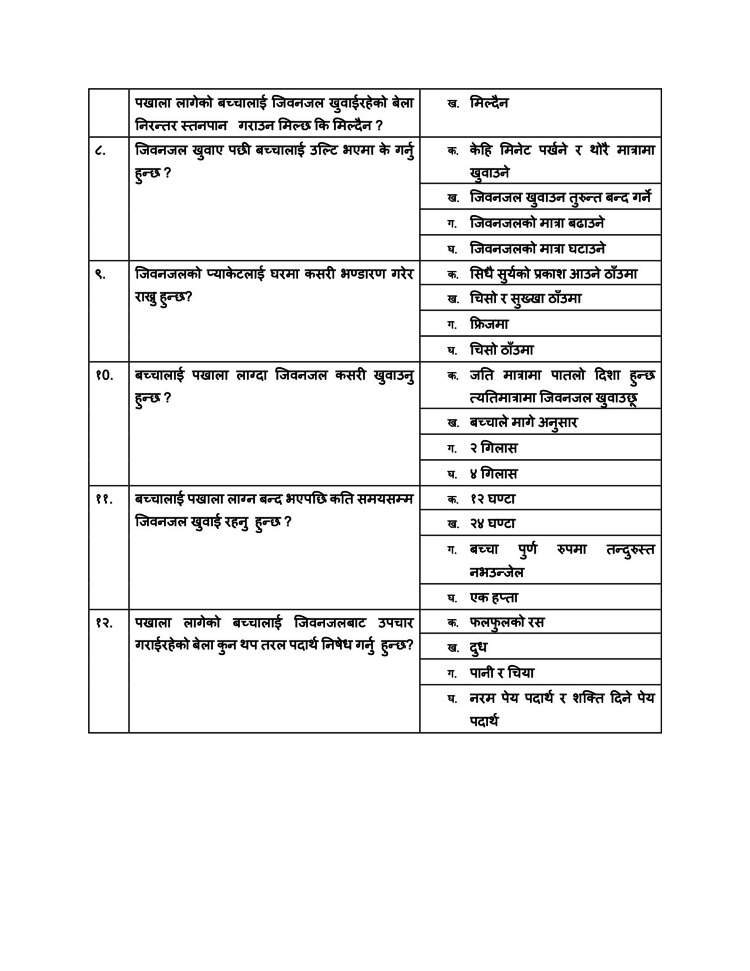
Nepali version of the questionnaire (continuation)
